# Impact of Admission of Patients With Syncope in Non-Teaching Hospitals Versus Teaching Hospitals: A Nationwide Analysis

**DOI:** 10.7759/cureus.39545

**Published:** 2023-05-26

**Authors:** Mushrin Malik, Garry Francis-Morel

**Affiliations:** 1 Internal Medicine, St. Barnabas Hospital Health System, New York City, USA

**Keywords:** gender comparison, absolute mortality, lenght of hospitalization, prevention of syncope, near syncope

## Abstract

Syncope is a common condition affecting many individuals, and it remains uncertain whether admission to academic medical centers (AMCs) leads to better outcomes than non-AMCs. This study is aimed to investigate whether there is a difference in mortality, length of stay (LoS), and total hospital charges between patients admitted with syncope to AMCs and non-AMCs. This retrospective cohort study used the National Inpatient Database (NIS) to examine patients aged 18 years and older admitted with a primary diagnosis of syncope to AMCs and non-AMCs from 2016 to 2020. Univariate and multivariate logistic regression analyses were conducted, adjusting for confounders, to assess the primary outcome of all-cause in-hospital mortality and secondary outcomes, including hospital LoS and total cost of admission. Patient characteristics were also described. Of the 451,820 patients who met the inclusion criteria, 69.6% were admitted to AMCs and 30.4% to non-AMCs. Patient age was similar between the two groups (68 years in AMC versus 70 years in non-AMC; p < 0.001), as was sex distribution (52% female in AMC versus 53% in non-AMC; 48% male in AMC versus 47% in non-AMC; p < 0.002). Most patients in both groups were white, while the percentages of black and Hispanic patients were slightly higher in non-AMCs. The study found no difference in all-cause mortality between patients admitted to AMCs and non-AMCs (p = 0.33). However, LoS was marginally longer in AMC patients (2.6 days in AMC versus 2.4 days in the non-AMC group; p < 0.001), and the total cost was higher for AMCs by $3,526 per admission. The estimated total economic burden related to syncope was over 3 billion USD per year. This study suggests that the teaching status of hospitals did not significantly affect the mortality of patients admitted with syncope. However, it may have contributed to marginally longer hospital LoS and higher total hospital charges.

## Introduction

Syncope is a transient loss of consciousness (TLoC) resulting from decreased cerebral blood flow [1]. While it can present in different ways, determining if the underlying etiology is cardiac is crucial since this group of patients has a worse prognosis [2]. The lifetime prevalence of syncope in the US is estimated to be 40%, with 1-3% of ED visits and 6% of hospital admissions resulting from syncope [3,4]. Despite the current consensus that history taking, physical examination, and EKG assessment are the cornerstones of syncope evaluation [3,5-7], risk stratification can be challenging [8-10], leading to unnecessary testing or admissions [11,12]. This challenge can make it difficult for physicians to balance the appropriate use of healthcare with identifying high-risk individuals for adequate management. Patients with syncope often undergo advanced imaging, significant consultation time, and hospital admission, leading to a significant economic burden on healthcare systems [2,13-15]. It has been estimated that the annual cost of syncope admission was close to $2.4 billion in the past, but no recent data are currently available [14, 16]. Furthermore, while there is evidence that academic medical centers (AMCs) incur higher expenses that might be justified by better outcomes, especially in patients with cancer [17-19], it is unclear whether admission to AMCs improves outcomes such as mortality in patients admitted for syncope [20]. In medical practice, it is essential to understand the quality of care (QoC) and cost. Despite this need, no recent study representative of the US population nationwide has addressed these questions. Therefore, we aimed to study the differences in outcomes, specifically mortality, length of stay (LoS), and total hospital cost, in patients admitted to AMC compared to those admitted to non-AMCs, to better understand the ratio of QoC and resource utilization. This study used the National Inpatient Sample (NIS) database to produce national estimates.

## Materials and methods

Study cohort

This study utilized the NIS database, the largest publicly available inpatient database in the United States. The sample included patients admitted between January 2016 and December 2020 with a primary diagnosis of syncope and collapse, as indicated by the International Classification of Diseases 10th Revision (ICD-10) code R55. Patients aged 18 years and older admitted to acute care facilities were included, while those with elective admissions and those younger than 18 years were excluded. The database did not include skilled nursing and long-term acute care facilities. The study cohort was divided into two groups based on the hospital's teaching status: AMCs and non-AMCs. An AMC was defined as a hospital accredited by the Accreditation Council for Graduate Medical Education (ACGME), a member of the Council of Teaching Hospitals (COTH), or any hospital with a ratio of full-time equivalent interns and residents to beds greater than 0.25 [21].

Study outcomes

The primary outcome of this retrospective cohort study was in-hospital all-cause mortality, which was defined as death during hospitalization and was coded in the database. This variable was not reported in 0.02% of hospitalizations, and admissions with missing data were excluded from the study. The secondary outcomes were hospital LoS and total hospital charge. LoS was defined as the total number of days from admission to discharge or death and was reported in 100% of hospitalizations. Total hospital charges were also coded in the database and were not reported in 0.8% of hospitalizations; similarly, hospitalizations with missing values were excluded from the sample. This variable was defined as the total hospital charges and reported in USD. General demographics, such as age, sex, and race, were recorded. This study was exempted from Institutional Review Board (IRB) review as it used data from the Healthcare Cost and Utilization Project (HCUP) databases, which are classified as limited datasets. Under the Health Insurance Portability and Accountability Act of 1996 (HIPAA) privacy rule, specified in 45 CFR, such limited datasets are exempt from IRB review.

Statistical methods

The statistical analysis was conducted using STATA MP/17 version (StataCorp, College Station, Texas, USA). The total number of patients that met the inclusion criteria was extracted, and baseline characteristics on patient demographics, hospitalization, and hospital characteristics were described. Differences between categorical variables, such as mortality, were tested using the Chi-square test. For hypothesis testing of secondary outcomes, a student's t-test was conducted for continuous variables. To account for confounders and effect modifiers, multivariate logistic regression analysis was performed, adjusting for age, race, income, hospital characteristics, and burden of comorbidity determined by the Charlson comorbidity index. Multivariate linear regression was used to determine predictors of LoS and resource utilization. Statistical significance was defined as a two-tailed p-value <0.05.

## Results

Patient characteristics

We analyzed data collected from the NIS database. We found 451,820 hospitalizations under the primary diagnosis of syncope and collapse over five years in the US, of which 69.6% were treated in AMCs, and 30.4% were treated in non-AMCs. The adjusted incidence rate of syncope using the United States Census Bureau was 0.34 per 1,000 person-years. Among all the hospitalized patients, the mean age was 69, with the mean age in non-teaching hospitals being 70 and in teaching hospitals being 68 (p <0.001). There was no clinically relevant difference in sex distribution (males: 47% in non-AMC versus 48% in AMC and females: 53% in non-AMC versus 52% in AMC; p = 0.002). White patients were higher in non-teaching hospitals, comprising 73.04% of the patient population, compared to 60.6% in teaching hospitals (p <0.001). In contrast, more Black patients were treated in teaching hospitals (21.08%) than non-teaching hospitals (12.53%). Additionally, the proportion of Hispanic patients was higher in non-teaching hospitals at 8.92%, compared to 10.94% in teaching hospitals (p <0.001). The proportion of patients who identified as Asian was similar between the two hospital types, with 2.85% in non-teaching hospitals and 2.96% in teaching hospitals. Similarly, the percentage of Native American patients was lower and similar between the two groups, with 0.4% in non-teaching hospitals and 0.31% in teaching hospitals (Table 1).

**Table 1:** Patient characteristics.

	All patients	Non-teaching	Teaching	P-value
Total	451,820	30.4	69.6	<0.001
Age	69.19	70.5	68.5	<0.001
Sex				0.002
Male	47.74	47	48	
Female	52.26	53	52	
Race				<0.001
White	64.38	73.04	60.6	
Black	18.47	12.53	21.08	
Hispanic	10.32	8.92	10.94	
Asian/PI	2.93	2.85	2.96	
Native American	0.34	0.4	0.31	
Others	3.56	2.26	4.11	
Median Household income				<0.001
$1-$49,999	30.54	32.53	29.67	
$50,000-$64,999	25.01	29.24	23.16	
$65,000-$85,999	23.59	21.82	24.37	
$86,000 or more	20.86	16.41	22.8	
Insurance				<0.001
Medicare	68.78	72.58	67.11	
Medicaid	10.83	8.62	11.8	
Private	16.95	15.37	17.64	
No insurance	3.44	3.43	3.45	
Hospital Region				<0.001
Northeast	26.4	13.77	31.94	
Midwest	18.27	18.86	18.01	
South	39.24	47.17	35.77	
West	16.09	20.2	14.29	
Hospital bed size				<0.001
Small	21.18	14.93	23.92	
Medium	31.58	30.78	31.93	
Large	47.24	54.29	44.15	
Total charges	35,242.04	32,798.60	36,323.99	<0.001
LOS	2.6	2.44	2.67	<0.001
Mortality	1135 (0.25)	0.28	0.24	0.33

In-hospital mortality

Among the 451,820 hospitalizations, 1,135 of them died. The overall mortality rate was 0.25%. The results suggest that age, gender, Charlson Index, and Hispanic race are significantly associated with mortality. Specifically, for each year's increase in age, the odds of mortality increased by a factor of 1.04 (95% CI: 1.02-1.05), and being female decreased the odds of mortality by a factor of 0.69 (95% CI: 0.52-0.91). A higher Charlson Index was associated with higher odds of mortality (aOR: 1.37, 95% CI: 1.30-1.44). In terms of race, Hispanic patients had significantly lower odds of mortality (aOR: 0.56, 95% CI: 0.31-1.01) compared to White patients. However, this association was only marginally significant, with a p-value of 0.05. There was no significant association between mortality and being Black, Asian/PI, Native American, or other race. The other predictors tested, including the teaching status of the hospital, median household income, insurance status, and hospital bed size, were not significantly associated with mortality, as shown in Table 2.

**Table 2:** Predictors of mortality. aOR: Odds of mortality.

	aOR	95% CI	P-value
Teaching Status	0.89	0.66-1.20	0.46
Age	1.04	1.02-1.05	<0.001
Female	0.69	0.52-0.91	0.01
Race			
White	Ref	Ref	Ref
Black	0.79	0.54-1.14	0.21
Hispanic	0.56	0.31-1.01	0.05
Asian/PI	0.91	0.42-1.96	0.82
Native American	1	-	-
Others	0.53	0.20-1.41	0.2
Median Household income			
$1-$49,999	Ref	Ref	Ref
$50,000-$64,999	0.89	0.62-1.29	0.56
$65,000-$85,999	0.95	0.65-1.38	0.81
$86,000 or more	0.81	0.54-1.22	0.33
Charlson Index	1.37	1.30-1.44	<0.001
Insurance			
Medicare	Ref	Ref	Ref
Medicaid	1.86	1.05-3.27	0.03
Private	1.09	0.64-1.85	0.74
No insurance	0.87	0.21-3.58	0.85
Hospital bed size			
Small	Ref	Ref	Ref
Medium	1.12	0.76-1.64	0.55
Large	1.02	0.70-1.49	0.88

Length of stay

The difference in LoS between patients admitted to AMCs and those not admitted to AMCs was found to be statistically significant but not clinically relevant (non-AMC: 2.44 days versus AMC: 2.67 days; p <0.001). Several factors were identified as positive predictors of extended in-hospital stay, including age (Coef 0.011, p <0.001), female sex (Coef 0.060, p = 0.001), black patients (Coef 0.224, p <0.001), and Charlson Comorbidity Index (CCI) (Coef 0.190, p <0.001). In addition, hospital bed sizes from medium (Coef 0.087, p = 0.006) to large (Coef 0.223, p <0.001) were also associated with an increased LoS. Conversely, several factors were identified as negative predictors of the LoS, including Hispanic race (Coef -0.120, p <0.001), Asian race (Coef -0.135, p = 0.039), household incomes over $50,000, and primary payer being private (Coef -0.12, p <0.001) or no insurance (Coef -0.193, p <0.001). These findings are summarized in Table 3.

**Table 3:** Predictors of length of stay.

	Coefficient	95% CI	P-value
Teaching status	0.255	0.21 | 0.29	<0.001
Age	0.011	0.00 | 0.01	<0.001
Female	0.06	0.02 | 0.09	0.001
Race			
White	Ref	Ref	Ref
Black	0.224	0.16 | 0.28	<0.001
Hispanic	-0.12	-0.17 | -0.06	<0.001
Asian/PI	-0.135	-0.26 |-0.00	0.039
Native American	0.042	-0.22 | 0.31	0.75
Others	0.013	-0.33	0.87
Median Household income			
$1-$49,999	Ref	Ref	Ref
$50,000-$64,999	-0.068	-0.11 | -0.01	0.006
$65,000-$85,999	-0.102	-0.15 | -0.04	<0.001
$86,000 or more	-0.187	-0.24 | -0.12	<0.001
Charlson Index	0.19	0.17 | 0.20	<0.001
Insurance			
Medicare	Ref	Ref	Ref
Medicaid	0.272	0.14 | 0.40	<0.001
Private	-0.12	-0.17 | -0.06	<0.001
No insurance	-0.194	-0.28 | -0.10	<0.001
Hospital bed size			
Small	Ref	Ref	Ref
Medium	0.087	0.02 | 0.14	0.006
Large	0.223	0.17 | 0.27	<0.001

Total hospital charges

The results of this study indicate a significant difference in hospital charges between AMCs and non-AMCs. The average charge for syncope patients at AMCs was $36,323, $3,525 higher than the average charge of $32,798 at non-AMCs (p < 0.001). As most patients were admitted to the AMC, this charge difference could account for a total cost differential of $1,383,504,003 annually. Therefore, the total cost of syncope nationwide was $3,185,546,66 per year. Moreover, the study found that certain demographic groups were more likely to incur higher charges per admission. Black (Coef 1,200; p = 0.005), Hispanic (Coef 7,823; p < 0.001), and Asian (Coef 6,965; p < 0.001) patients were associated with higher charges. Additionally, a median household income of over $86,000 (Coef 2,710; p < 0.001) was found to be a positive predictor of the total cost (p < 0.001). Furthermore, the CCI (Coef 1,566, p < 0.001) and hospital bed sizes of medium (Coef 3,983; p < 0.001) and large (Coef 4,078; p < 0.001) were also significantly associated with higher costs. The results are presented in Table 4.

**Table 4:** Predictors of total cost.

	Coefficient	95% CI	P-value
Teaching Status	3,171.58	2249 | 4093	<0.001
Age	-11.41067	-31 | 8.74	0.26
Female	-470.7878	-915 | -25	0.03
Race			
White	Ref	Ref	Ref
Black	1,200.81	358 | 2024	0.005
Hispanic	7,823.40	6589 | 9057	<0.001
Asian/PI	6,965.25	4443 | 9487	<0.001
Native American	-3,829.77	-7784 | 125	0.058
Others	4,614.47	2360 | 6868	<0.001
Median Household income			
$1-$49,999	Ref	Ref	Ref
$50,000-$64,999	-45.35869	-827 | 736	0.9
$65,000-$85,999	421.8933	-468 | 1312	0.35
$86,000 or more	2,710.64	1558 | 3862	<0.001
Charlson Index	1,566.15	1418 | 1713	<0.001
Insurance			
Medicare	Ref	Ref	Ref
Medicaid	-1,168.14	-2255 | -80	0.035
Private	-301.1065	-1059 | 457	0.43
No insurance	-1,594.52	-2902 | -287	0.017
Hospital bed size			
Small	Ref	Ref	Ref
Medium	3,983.27	2813 | 5153	<0.001
Large	4,078.99	2989 | s5168	<0.001

## Discussion

Teaching hospitals in the United States are renowned for providing high-quality care [17, 20]. A literature review conducted in 2002 concluded that the quality of care in teaching hospitals is superior to that in non-teaching hospitals for common conditions among elderly patients [22]. Moreover, when we think about common conditions, we cannot disregard syncope, as it affects about one in three adults during their lifetime [15]. As we sought to investigate the impact of teaching status on syncope admissions, we found that about 90,364 hospitalizations occur annually in the US. The incidence rate, adjusted using the United States Census Bureau data, was 0.34 per 1,000 person-years. While this represents a decrease in frequency compared to the initial Framingham Heart Study data [23], it is consistent with recent data suggesting that the incidence is between 0.80 and 0.93 per 1,000 person-years [24]. Our results demonstrate that more patients are admitted to teaching hospitals with teaching status, with 69.6% admitted to teaching hospitals compared to 30.4% admitted to non-teaching hospitals (p <0.001). This is likely due to teaching hospitals' better reputation for providing quality healthcare [17]. However, contrary to previous studies [20], the teaching status of the hospitals did not show any statistically significant impact on mortality (p = 0.33). While this finding might seem counterintuitive, we hypothesized that it could partly be explained by the low overall mortality associated with this condition. Death associated with syncope is uncommon and generally limited to patients with underlying structural heart diseases [25]. 

We observed a statistically significant difference in the LoS (p <0.001) and total cost (p <0.001) of patients admitted to teaching hospitals with syncope. The effect of a hospital's teaching status on syncope-related hospital admissions has not been extensively studied, and we lack a concrete explanation. However, a literature review on the prognosis of patients admitted to teaching hospitals suggests that patients treated at teaching hospitals and high-volume centers tend to be sicker and frequently have complex clinical conditions involving different organ systems compared to those treated in non-teaching hospitals [26, 27]. As a result, patients require more extensive work, resulting in longer LoS and increased costs in teaching hospitals. In a study by Sloan FA, the approximate hospital cost was observed to be at most 20% higher in major teaching hospitals than in non-teaching hospitals, but their study was based on a sample of 367 hospitals [17]. Therefore, it is assumed that the increased cost is due to examining cost differences by cost center, such as higher costs for investigations and pathological tests at major teaching hospitals.

The cost of hospital care is generally higher in teaching hospitals than in non-teaching hospitals due to factors such as higher staff costs and more complex medical cases [28]. Our study estimated the annual in-hospital cost of syncope nationwide to be over $3 billion per year, which is higher than previously reported [14].

Age was found to be a significant predictor of mortality (adjusted odds ratio [aOR]: 1.04, p <0.001) and increased LoS (p <0.001) in our analysis. Syncope is typically caused by defects in blood pressure regulatory mechanisms, such as the cardiovascular system, baroreceptor function, autonomic nervous systems, and humoral systems, which are more common in older adults [12]. Additionally, dehydration is a significant cause of orthostatic syncope, and older individuals are more susceptible to dehydration due to reduced thirst sensation and alterations in renal function [29-31]. The prevalence of syncope increases with age, with the highest incidence observed in patients aged 70-89 years [23].

Our study also found that female patients had higher rates of hospital admissions than males and were less likely to experience mortality as an outcome in both univariate and multivariate models, with lower total costs. However, females were associated with increased LoS. Reflex syncope, the most common cause of syncope, is more prevalent in women, with more episodes of reflex syncope in women than men (7.2 ± 9.4 vs. 5.0 ± 6.4, p = 0.001), which may explain the increased admission rates [32]. 

In our analysis of the effect of race on outcomes in syncope-related hospital admissions, we found that mortality did not differ significantly between white and non-white patients in the univariate regression model. However, the Black race was associated with increased LoS compared to the White race, while the Hispanic and Asian races had decreased LoS compared to the White race. Furthermore, the total cost of care was higher for Black, Hispanic, and Asian races than for White races, with Hispanics having the highest total cost. In contrast, Native Americans had lower total costs compared to White Americans. Despite a thorough literature review, we could not identify any previous studies analyzing the impact of race on syncope-related admissions. While these findings are difficult to explain, they are essential for guiding the management of patients with syncope.

Study limitations

The data used in this study was obtained from the NIS database, which has its own limitations. The NIS database does not track individuals but rather inpatient stays, thus limiting our ability to identify if the same individual was admitted multiple times. This, in turn, may overestimate the sample size as the study analyzed a five-year cohort. Additionally, as randomization cannot be performed in observational studies, we could not account for selection bias or residual confounders. Nonetheless, it would be ethically inappropriate to assign patient admissions to an AMC or non-AMC to assess the impact of mortality.

## Conclusions

Our study analyzed a large cohort of over 400,000 patients over a period of five years and found that approximately 0.24-0.28% of admitted patients died during their in-hospital stay. Interestingly, we found no evidence suggesting that admitting patients with syncope to AMC improved mortality outcomes compared to non-AMC. While prior research had suggested that some medical conditions may have better outcomes when treated in AMC, this did not hold true for syncope patients. Moreover, the independent mortality predictors were age, the burden of comorbidities assessed using CCI, and Medicaid as the primary payer. LoS was found to be only marginally increased by 0.2 days, which may be clinically insignificant. Additionally, we found that admissions to AMC incurred costs that were 3,526 USD higher than those to non-AMC. Overall, our study estimates the economic burden of syncope to be over three billion dollars annually.
